# Preclinical evaluation of recombinant type III collagen-based contact lens rewetting drops

**DOI:** 10.3389/fopht.2026.1861277

**Published:** 2026-08-12

**Authors:** Siyang Liu, Yefei Shi, Yuanxin Zhai, Jie Chen, Hanzhuang Teng, Jie Li, Yujie Meng, Jing Li, Shaohui Pan, Haihang Li, Shuxian Hu

**Affiliations:** 1 Jiangsu Trautec Medical Technology Co., Ltd, Changzhou, China; 2 Anhui Trautec Medical Technology Co., Ltd, Suzhou, China; 3 Eye Research Center, Hangzhou Institute of Medicine, Chinese Academy of Sciences, Eye Hospital, Wenzhou Medical University, Hangzhou, China; 4 State Key Laboratory of Eye Health, Eye Hospital, Wenzhou Medical University, Wenzhou, China; 5 Zhejiang Key Laboratory of Key Technologies for Visual Pathway Reconstruction, Eye Hospital, Wenzhou Medical University, Wenzhou, China; 6 State Key Laboratory of Digital Medical Engineering, School of Biological Science and Medical Engineering, Southeast University, Nanjing, China

**Keywords:** biomaterial, contact lens, recombinant collagen, rewetting drops, safety, Type III collagen

## Abstract

**Purpose:**

To evaluate the physicochemical properties, contact lens compatibility, *in vitro* cytocompatibility, and repeated-dose ocular safety of a recombinant type III collagen-based contact lens rewetting drop formulation (Col RD).

**Methods:**

Physicochemical properties were characterized using rheological testing and contact angle measurements. Total residual protein on rigid gas-permeable contact lenses was evaluated using an artificial tear fluid model, followed by non-enzymatic and enzymatic cleaning procedures. *In vitro* cytocompatibility was assessed using human corneal epithelial cells, including cell viability, adhesion, and migration assays. A one-month repeated-dose ocular safety study was conducted in New Zealand rabbits. Outcomes included slit-lamp examination, fluorescein staining, hematological and serum immunological analysis, and histopathological evaluation.

**Results:**

Col RD exhibited shear-thinning rheological behavior and a hydrophilic sequence profile, with viscosity close to that of human tears under blinking-associated shear conditions. Contact angle analysis showed no significant differences compared with commercial lubricants. In the *in vitro* rigid gas-permeable lens model, Col RD did not increase total residual protein after enzymatic cleaning and showed significantly lower post-cleaning residual protein than the hyaluronic acid-based comparator under the tested conditions. *In vitro*, Col RD showed no significant adverse effects on corneal epithelial cell viability, adhesion, or migration compared with controls. *In vivo*, no evident ocular irritation, epithelial damage, inflammatory response, or structural abnormality was observed during the 30-day administration period. Hematological and serum immunological parameters showed no significant differences among groups. Histological analysis revealed no necrosis, fibrosis, neovascularization, or abnormal collagen deposition. Sirius Red showed no evident alteration in type I/III collagen distribution.

**Conclusions:**

Recombinant collagen-based contact lens rewetting drops showed favorable rheological properties, acceptable *in vitro* cytocompatibility, and no evident ocular irritation after repeated topical administration in rabbits. In an *in vitro* rigid gas-permeable lens model, Col RD showed low total residual protein after enzymatic cleaning compared with the hyaluronic acid-based formulation. These findings support the preliminary safety and feasibility of incorporating recombinant type III collagen into a PVA-containing lubricating formulation for contact lens rewetting drops. Further studies are needed to evaluate clinical rewetting performance, broader lens-material compatibility, long-term formulation stability, and the relative contribution of recombinant collagen and PVA.

## 1 Introduction

With the convenience of contact lenses and the popularization of orthokeratology lenses in the prevention and control of myopia in adolescents, the number of people using contact lenses is increasing day by day (1, 2). Due to the widespread use of contact lenses, the associated complications are also increasing. Long-term wearing of contact lenses easily causes the accumulation of deposits on the inner surface of the lenses (3). As a foreign body, contact lenses will affect and irritate the eyelid margin, leading to excessive secretion of the meibomian glands, thereby increasing the deposition on the lens surface (4). In this case, the tear fluid cannot normally lubricate the lens surface; instead, it causes increased eye discomfort, dryness, foreign body sensation, and blurred vision. Therefore, as an important supporting product, the development of contact lens rewetting drops has attracted increasing attention. In the presence of the above conditions, instillation of rewetting drops can greatly improve eye discomfort, effectively relieve visual fatigue, and reduce friction between the eye, eyelid, and lens (5). Contact lens rewetting drops are commonly used eye care products for contact lens wearers, and their safety is directly related to ocular health (6). Although sodium hyaluronate-, polyvinyl alcohol-, and carboxymethyl cellulose -based rewetting formulations are widely used, the interaction between polymeric lubricants, tear proteins, and contact lens materials remains formulation- and material-dependent (6–8). Therefore, alternative biomimetic lubricating ingredients with acceptable ocular safety and lens compatibility remain of interest.

Collagen has broad application prospects in the field of eye care due to its good biocompatibility, moisturizing properties, and biological activity (9, 10). Type III collagen is a fibrillar extracellular matrix collagen encoded by COL3A1. It is widely distributed in mechanically compliant and extensible connective tissues and frequently coexists with type I collagen in heterotypic fibrillar matrices. Previous studies have shown that type III collagen is more closely associated with flexible matrix organization, early matrix remodeling, and regulation of collagen fibril architecture (11, 12). In normal corneal stroma, type III collagen is weakly expressed; however, its expression increases after stromal injury or during corneal wound healing, suggesting that type III collagen participates in the repair-associated extracellular matrix response (13). This biological context provides a rationale for considering type III collagen as a biomimetic component for ocular surface applications. Previous studies have investigated recombinant human type I and type III collagen hydrogels as tissue-engineered corneal substitutes (14–17). These materials showed good transparency, mechanical robustness, epithelial and nerve compatibility *in vitro*, and acceptable performance after implantation in animal models, supporting the broader feasibility of recombinant human collagen, including type III collagen, in ophthalmic biomaterials.

Therefore, recombinant type III collagen was selected in this study as a human-derived, hydrophilic, and ocular-biomaterial-relevant protein component, and its suitability was evaluated through physicochemical characterization, contact lens residue testing, *in vitro* cytocompatibility, and repeated-dose ocular safety assessment. Importantly, the present formulation was not designed to replace native corneal collagen or to induce corneal tissue regeneration, but to use a human-sequence-derived recombinant type III collagen fragment as a hydrophilic protein-based lubricating ingredient for contact lens rewetting drops. The study was designed as a preclinical safety and feasibility evaluation rather than a clinical efficacy study.

## 2 Materials and methods

### 2.1 RRecombinant collagen and recombinant collagen rewetting drops

Recombinant collagen was derived from the amino acid sequence of the α1 chain of natural human recombinant collagen and obtained through Pichia pastoris recombinant fermentation technology and purification, provided by Jiangsu Trautec Medical Technology Co., Ltd. The recombinant collagen used in this study was designed based on a selected fragment of the human type III collagen α1 chain. The recombinant sequence contains 500 amino acids, with a molecular weight of approximately 44 kDa and an isoelectric point of 9.1. No exogenous affinity tag or non-human functional domain was introduced into the construct. The short terminal sequences GPP and GG were included at the N- and C-termini, respectively, to protect terminal structure and reduce terminal heterogeneity, rather than to serve as exogenous tags or linker peptides.

Collagen rewetting drops consisted of 1% reCol and 0.5% PVA (Sichuan Juxin Development Technology, China), dissolved in phosphate buffer (Xi’an Tianzheng Pharmaceutical Excipients, China). The detailed formulation and properties of Col RD are shown in **Table 1**.

**Table 1:** The formulation and properties of Col RD.

Item	Parameters
Recombinant type III collagen	1% w/w
PVA	0.5% w/w
Na_2_HPO_4_	0.3% w/w
NaH2PO_4_·H2O	0.3% w/w
NaCl	0.65% w/w
Sterilization/aseptic processing	0.22 μm filtration/aseptic filling
Appearance	clear, colorless solution
pH	7.0
Osmolality	301 mOsm/kg
Preservative	preservative-free
Endotoxin	<0.05 EU/mL

### 2.2 Experimental animals

Nine New Zealand rabbits were used in this study, including 5 females and 4 males, randomly grouped using a random-number table. As experimental animal models, the rabbits were housed in the animal laboratory of Guangzhou Huateng Biomedical Technology Co., Ltd. under conventional conditions. The laboratory animal use license number was SYXK (Yue) 2024-0307 (valid until January 29, 2029, issued by the Guangdong Provincial Department of Science and Technology). Animals were housed in stainless steel single cages. The environmental temperature was maintained at 18–26 °C, with a daily temperature difference not exceeding 4 °C, relative humidity of 40–70%, and a 12 h light/12 h dark cycle. All animals used in this study had undergone at least 7 days of quarantine and environmental acclimatization. At the end of the observation period, rabbits were deeply anesthetized by intramuscular injection of Sumianxin II at a dose of 0.2 mL/kg. After confirmation of deep anesthesia, animals were euthanized by exsanguination through abdominal aorta bleeding, and tissues were collected for subsequent histopathological evaluation. The animal study was reported in accordance with the ARRIVE guidelines where applicable.

### 2.3 Main reagents and instruments

Recombinant collagen (Trautec Medical Technology, China), saline (Guangdong Mingkang Medical Equipment, China), polyvinyl alcohol (Sichuan Juxin Development Technology, China), sodium bicarbonate (Aladdin, China), sodium dihydrogen phosphate (Aladdin, China); Rigid Gas Permeable Contact Lensesfor Orthokeratology (Eyebright Medical, China), Optive contact lens rewetting drops (Santen Pharmaceutical Co., Ltd.); rigid gas permeable lens rinsing solution (Euclid Systems Corporation); Ximing multifunctional rigid contact lens care solution (Eyebright Medical Technology Co., Ltd.); Shuming rigid contact lens enzymatic cleaner (Eyebright Medical Technology Co., Ltd.); trifluoroacetic acid (Hushi), acetonitrile (Adamas), sodium chloride (Jiangsu Qinfen Pharmaceutical Co., Ltd.), potassium chloride (Macklin), calcium chloride (Adamas), magnesium chloride (Aladdin), albumin (Sigma-Aldrich), lysozyme (Sigma-Aldrich), oleic acid (Adamas), palmitic acid (Adamas), lauric acid (Adamas), hexadecyl tetradecanoate (Adamas), n-hexadecane (Solarbio), squalene (Adamas), cholesterol (Adamas), bovine type I collagen (Sigma-Aldrich), DMEM/F12, FBS, human EGF (Gibco), MTT Cell Proliferation and Cytotoxicity Assay Kit (Biotime).

Spectrophotometer Varioskan LUX (Thermo Scientific), rheometer (HR10, Waters), scanning electron microscope (HITACHI, TM3030 PLUS), contact angle goniometer (SDC100S, SINDIN).

### 2.4 Characterization of rCol

#### 2.4.1 Hydrophilicity analysis

The hydrophilicity of the recombinant sequence was evaluated using the grand average of hydropathicity (GRAVY), a sequence-based parameter calculated as the sum of the hydropathy values of all amino acid residues divided by the sequence length (18). A lower or more negative GRAVY value generally indicates greater overall hydrophilicity.

#### 2.4.2 Scanning electron microscopy

SEM was used to observe the solid-state morphology of lyophilized recombinant collagen and to provide descriptive information on its microstructural appearance. For SEM analysis, the cross-section of recombinant collagen samples was fixed onto a sample stage using conductive adhesive. The sample edges were additionally bridged with conductive adhesive to enhance electrical conductivity. A gold coating was applied to the sample surface via sputter coating for 90 seconds to improve conductivity and imaging quality. Microstructural characterization was performed using SEM under an accelerating voltage of 15 kV and a working distance of 15 mm.

#### 2.4.3 Gel electrophoresis

SDS-PAGE was performed to assess the apparent molecular weight distribution and electrophoretic purity of recombinant collagen. A 10 mg/mL recombinant collagen aqueous solution was prepared. An appropriate amount of ddH_2_O and 5× loading buffer were added, mixed thoroughly, heated in boiling water for 5 min, and then centrifuged. Samples and molecular weight markers were loaded into the gel wells. The gel apparatus was assembled and connected to electrodes. Electrophoresis was run at 80 V for 15 min, followed by 120 V for 1 h. The gel was then removed, stained, and imaged.

#### 2.4.4 Peptide coverage analysis

LC-MS/MS peptide mapping was used to confirm the sequence coverage and identity of the recombinant collagen construct. Samples were first subjected to reduction and alkylation, followed by SP3-based enrichment. SP3, a single-pot solid-phase-enhanced sample-preparation method, was used for protein cleanup and peptide recovery before LC-MS/MS analysis. Enzymatic digestion was then performed using trypsin, trypsin + Glu-C, and Glu-C. Glu-C is an endoproteinase that preferentially cleaves peptide bonds C-terminal to glutamic acid residues and was used together with trypsin to improve peptide coverage. The processed samples were analyzed by liquid chromatography–tandem mass spectrometry (LC-MS/MS). Raw data files were obtained and analyzed using Thermo BioPharma Finder software for peptide identification.

LC conditions: Mobile phase A, water with 0.1% formic acid; Mobile phase B, 80% acetonitrile with 0.1% formic acid. Gradient: 0–2 min, 4-8%B;2-35min, 8-28%B;35-55min, 28-40%B;55-56min, 40-95%B;56-66min, 95-95%B. Flow rate: 600 nL/min. Column: 150 μm i.d. × 170 mm, packed with ReproSil-Pur 120 C18-AQ, 1.9 μm.

MS conditions: MS1 resolution: 70,000; AGC target: 3 × 10^6^; Maximum injection time: 100 ms; Scan range: 300–1800 m/z; MS2 resolution: 17,500; AGC target: 1 × 10^5^; Maximum injection time: 50 ms; Normalized collision energy (NCE): 28 (stepped NCE used).

### 2.5 Rheology

Rheological testing was performed to evaluate whether Col RD exhibited shear-dependent viscosity behavior relevant to blinking-associated shear conditions. A rheometer (cone-plate/parallel plate) was used to perform shear rate scanning. By applying a controllable shear rate, the viscosity versus shear rate curve (η) was obtained, reflecting the shear-dependent behavior of non-Newtonian fluids. During testing, 0.3 mL of sample was carefully placed at the center of the measuring geometry. A 40 mm cone plate was used, and the gap was set to 27 μm. Shear rate and viscosity scanning were performed at shear rates of 0.1–100 s^-^¹ at 25 °C, and the dynamic viscosity at a shear rate of 25 s^-^¹ was recorded.

### 2.6 Hydrophilicity analysis of Col RD

Contact angle analysis was used as a comparative solid-state wettability assay for lyophilized formulation residues, rather than as a direct measure of the aqueous drop behavior on the ocular surface. Based on the surface tension of liquids and the properties of solid surfaces, when a liquid contacts a solid, the properties of the solid surface affect the action of surface tension, thereby forming a contact angle. By comparing the contact angles among different samples, the hydrophilicity of collagen was evaluated. During testing, the samples were freeze-dried and compressed into tablets, 3 μL of pure water was dropped onto the surface, and the static contact angle was measured using the sessile drop method.

### 2.7 Residual protein on RGP lenses after simulated tear exposure and cleaning

Artificial tear solution was prepared, containing inorganic salts (sodium chloride 5.93 mg/mL, potassium chloride 2.35 mg/mL, calcium chloride 0.22 mg/mL, magnesium chloride 0.08 mg/mL, sodium bicarbonate 2.18 mg/mL, sodium dihydrogen phosphate 0.01 mg/mL); proteins (albumin 2.90 mg/mL, lysozyme 0.54 mg/mL); lipids (oleic acid 0.18 mg/mL, palmitic acid 0.006 mg/mL, lauric acid 0.05 mg/mL, hexadecyl tetradecanoate 0.10 mg/mL, n-hexadecane 0.05 mg/mL, squalene 0.07 mg/mL, cholesterol 0.07 mg/mL) (19).

Eighteen rigid gas permeable contact lenses were randomly divided into three groups (Col RD, HA RD, Saline) and placed in a 24-well plate. Then, 2 mL of artificial tear solution was added to each well. The plates were incubated in a constant temperature shaking incubator at 37 °C and 80 rpm for 12 h. During incubation, 20 μL of the corresponding rewetting drops (Col RD, HA RD, Saline) were added to each well every 2 h.

Three lenses from each group were taken as the enzymatic cleaning group (EH). The lens surface was first vortexed with 2 mL rigid contact lens rinsing solution for 30 s to remove easily removable proteins and impurities. The cleaned lenses were then immersed in 2 mL rigid contact lens care solution, 100 μL enzymatic cleaner was added, and the lenses were incubated overnight in a constant temperature shaking incubator at 37 °C and 80 rpm. Afterward, 2 mL rinsing solution was added again, followed by ultrasonication for 15 min. The lenses were rinsed with saline and air-dried. For the non-enzymatic cleaning group, no enzymatic cleaner was used. The lenses were only immersed overnight in rinsing solution at 37 °C and 80 rpm.

All lenses were then transferred into a new 24-well plate, and 2.0 mL of 0.2% trifluoroacetic acid–acetonitrile solution was added. The lenses were incubated at 37 °C and 80 rpm to elute the adsorbed proteins. The eluted solutions were collected, rapidly frozen at −80 °C, and then dried in a freeze dryer. Protein content was quantified using the NanoOrange^®^ protein assay kit (Thermo Scientific) according to the manufacturer’s instructions.

### 2.8 *In vitro* cytocompatibility assessment of Col RD

The cell viability, adhesion, and migration assays were performed to evaluate the *in vitro* cytocompatibility of Col RD with human corneal epithelial cells. These assays were used to determine whether Col RD adversely affected basic epithelial cell behaviors relevant to ocular surface safety.

HCE-T cells were purchased from Procell (CL-0743, Wuhan, China).For the cell proliferation assay, 100 μL of each test sample was added into 96-well plates and incubated at 37 °C with 5% CO_2_ for 4 h. Excess coating solution was removed from each well, and 100 μL of 1% BSA-PBS solution was added, followed by incubation at 37 °C with 5% CO_2_ for 1 h. After removing the liquid, the wells were washed three times with D-PBS, and the washing solution was discarded. A suspension of HCE-T cells (5.0 × 10^4^ cells/mL) was then added to the 96-well plates and cultured at 37 °C with 5% CO_2_. After 48 h of incubation, 50 μL of MTT solution (1 mg/mL) was added to each well, followed by further incubation for 2 h in the incubator. The supernatant was then discarded, and 100 μL of isopropanol was added to each well. The plate was shaken for 2 min, and absorbance was measured at 570 nm using a microplate reader (with a reference wavelength of 650 nm).

For the cell adhesion assay, 100 μL of the test solution was added to each well of a 96-well plate and incubated at 37 °C with 5% CO_2_ for 4 h. Excess coating solution was removed, and 100 μL of 1% BSA-PBS solution was added, followed by incubation at 37 °C with 5% CO_2_ for 1 h. After removal of the liquid, the wells were washed three times with D-PBS, and the wash solution was discarded. HCE-T cells were pre-mixed with Hoechst 33342 fluorescent dye (10%) in complete medium and diluted to 5 × 10^4^ cells/mL. Then, 100 μL of the cell suspension was added to each well. The plate was covered with aluminum foil and incubated at 37 °C with 5% CO_2_ for 1 h. Fluorescence images of each well were captured using an inverted fluorescence microscope. Each well was then filled with D-PBS to form a “reverse meniscus,” air bubbles were removed, and the plate was sealed. The plate was centrifuged upside down at the optimal relative centrifugal force at 22 °C for 5 min. After centrifugation, the sealing film was removed, and the supernatant was discarded. The wells were washed once with D-PBS, followed by the addition of 100 μL D-PBS. Each well was then imaged again. The adhesion rate was calculated as the ratio of cell number after centrifugation to the cell number before centrifugation.

For the cell migration assay, 2 mL of cell suspension (5.0 × 10^4^ cells/mL) was seeded into a 6-well plate and cultured at 37 °C with 5% CO_2_ for 24 h until the cells reached 95–100% confluence. A straight scratch was made using a 1000 μL pipette tip aligned vertically with a ruler. Cells were washed three times with PBS to remove detached cells. In the test and reference groups, 2 mL of serum-free medium containing the test or control material was added. In the blank control group, serum-free medium containing an equivalent volume of saline was added. Images were taken at 0 h and 24 h under a 40× microscope. The scratch area was analyzed using ImageJ software (v.1.53; National Institutes of Health, Bethesda, MD, USA). Cell migration rate was calculated as the ratio of the migrated area to the initial scratch area.

### 2.9 Repeated-dose ocular safety study in rabbits

A group-controlled experimental design was used. Animals were divided into the test group recombinant collagen rewetting drops (Col RD, n = 3), the control group hyaluronic acid rewetting drops (HA RD, n = 3), and the blank group (Saline, n = 3). All animals received bilateral ocular administration, three times daily at 0 h, 4 h, and 8 h. After each instillation, the eyelids were gently closed for 10–15 s to ensure full contact between the formulation and ocular tissues. The treatment was continuously administered for 30 days.

Gross observations were conducted on days 1, 14, and 30 after administration. After 30 days of dosing, hematological examination was performed, followed by euthanasia. Gross dissection was then carried out, and tissues were collected for histopathological examination.

Histopathological sections from all groups were examined microscopically and evaluated in a blinded manner to determine whether pathological damage was present at the examined site. When pathological changes were observed, the severity of the lesions was scored according to the criteria shown in **Table 2**.

**Table 2:** Histological evaluation: cell type/tissue reaction scoring criteria.

Item	Score 0	Score 1	Score 2	Score 3	Score 4
Polymorphonuclear cells	0	Minimal, 1–5/phf	5–10/phf	Severe infiltration	Full field
Lymphocytes	0	Minimal, 1–5/phf	5–10/phf	Severe infiltration	Full field
Plasma cells	0	Minimal, 1–5/phf	5–10/phf	Severe infiltration	Full field
Macrophages	0	Minimal, 1–5/phf	5–10/phf	Severe infiltration	Full field
Giant cells	0	Minimal, 1–2/phf	3–5/phf	Severe infiltration	Sheet-like distribution
Necrosis	0	Minimal	Slight	Moderate	Severe
Neovascularization	0	Mild capillary proliferation, focal, 1–3 vessels	4–7 groups of capillary proliferation, with fibroblast structures	Larger area of capillary proliferation, with fibroblast structures	Extensive capillary proliferation, with fibroblast structures
Fibrosis	0	Localized area	Moderate area	Thick area	Extensive area
Fatty infiltration	0	Very few adipocytes, accompanied by fibrosis	Several layers of adipocytes and fibrosis	Expanded area of adipocyte aggregation extending from the implantation site	Implant surrounded completely by adipocytes

phf = high-power field (400×).

### 2.10 Statistical analysis

Normality was assessed using the Shapiro–Wilk test. Normally distributed data were analyzed using one-way ANOVA followed by Tukey’s multiple comparisons test. Non-normally distributed data were analyzed using the Kruskal–Wallis test followed by Dunn’s multiple comparisons test. Data are presented as mean ± SD. A value of P < 0.05 was considered statistically significant (* p < 0.05, ** p < 0.01, and *** p < 0.001); ns, not significant. The data were analyzed using GraphPad Prism (v. 11.0.0; GraphPad Software Inc, La Jolla, USA).

## 3 Results

### 3.1 Physicochemical characterization of recombinant collagen

Hydrophilic amino acid composition is relevant to the intended lubricating and moisturizing function of the formulation, as hydrophilic protein segments may favor interactions with water molecules and contribute to aqueous compatibility. Therefore, the hydrophilicity of the recombinant sequence was evaluated using the grand average of hydropathicity (GRAVY), a sequence-based parameter calculated as the sum of the hydropathy values of all amino acid residues divided by the sequence length. The recombinant collagen sequence showed a GRAVY value of −1.011, which was lower than that of the full-length human type III collagen α1 chain (−0.797), indicating a relatively more hydrophilic sequence profile. This result is consistent with its intended use as a water-compatible protein component in an aqueous contact lens rewetting formulation.

**Figure 1:**
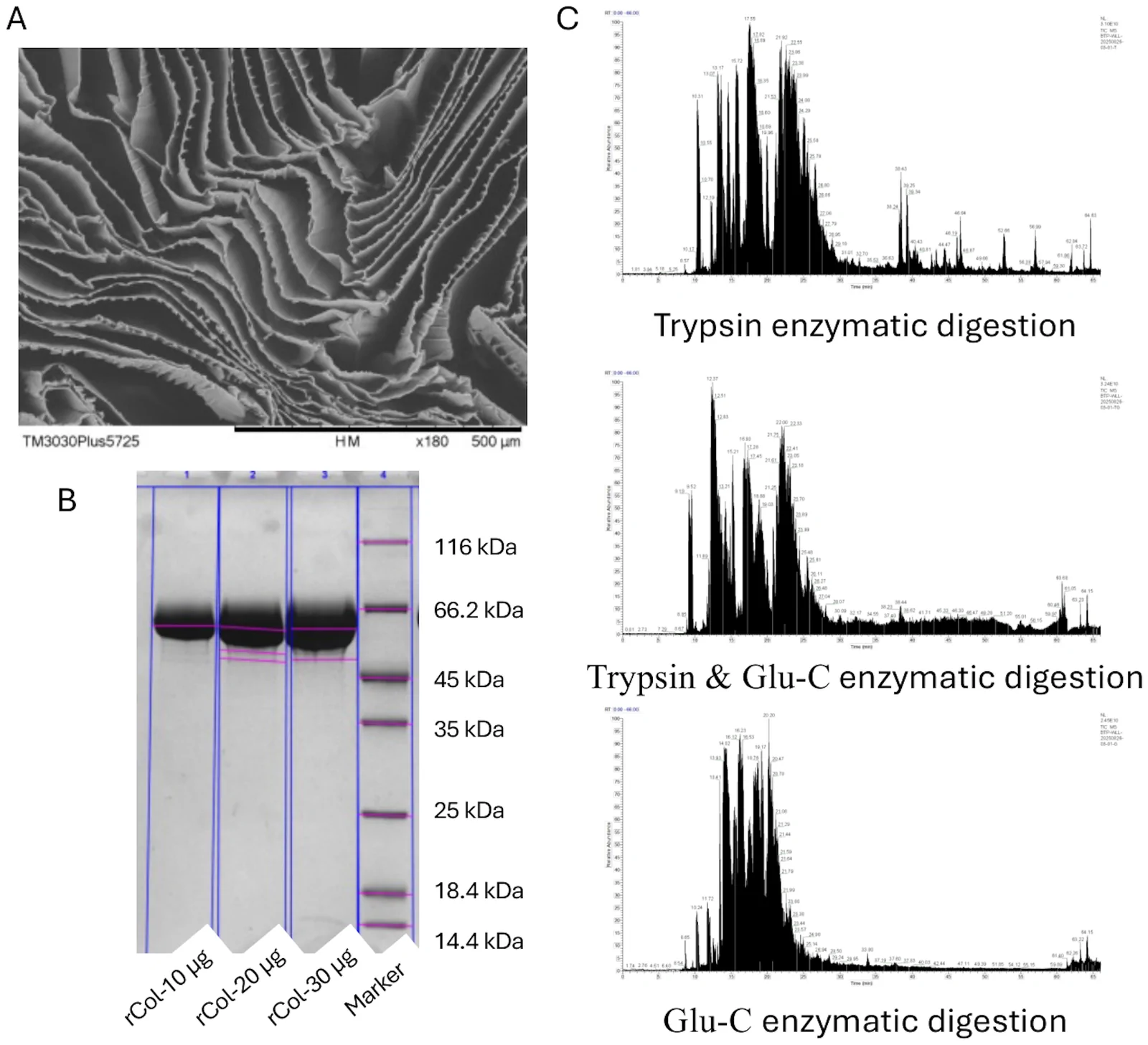
Characterization of Recombinant Collagen (rCol). **(A)** The rCol was observed to form a well-ordered lamellar structure analyzed by scanning electron microscopy (SEM). **(B)** The gel imaging system scanning after SDS-PAGE analysis. **(C)** LC-MS/MS spectra of rCol after enzymatic digestion with Trypsin, Trypsin & Glu-C, and Glu-C.

The rCol samples were analyzed by scanning electron microscopy (SEM), and the recombinant type III collagen was observed to form a well-ordered lamellar structure (**Figure 1A**). SDS-PAGE analysis followed by gel imaging system scanning showed that the main band average purity of the recombinant type III collagen sample was 93.54% (**Figure 1B**). After reduction and alkylation, the sample was subjected to enzymatic digestion using trypsin, trypsin & Glu-C, and Glu-C (**Figure 1C**). The resulting peptides were analyzed by liquid chromatography–tandem mass spectrometry (LC-MS/MS). Data processing and matching were performed using Thermo BioPharma Finder, and the results indicated a 100% sequence coverage of the target protein.

### 3.2 Dynamic viscosity and hydrophilicity of Col RD

The recombinant collagen sequence used in this study was derived from selected sequences of native human type III collagen, with preference given to fragments enriched in hydrophilic amino acids. No non-human-derived sequences or tag sequences were introduced. Computational simulations indicated that the obtained recombinant collagen sequence exhibited superior hydrophilicity compared with native type III collagen (20). By adding 0.5% (w/w) polyvinyl alcohol (PVA) and dissolving it in phosphate-buffered saline (PBS), a recombinant collagen rewetting drops (Col RD) was formulated.

**Figure 2:**
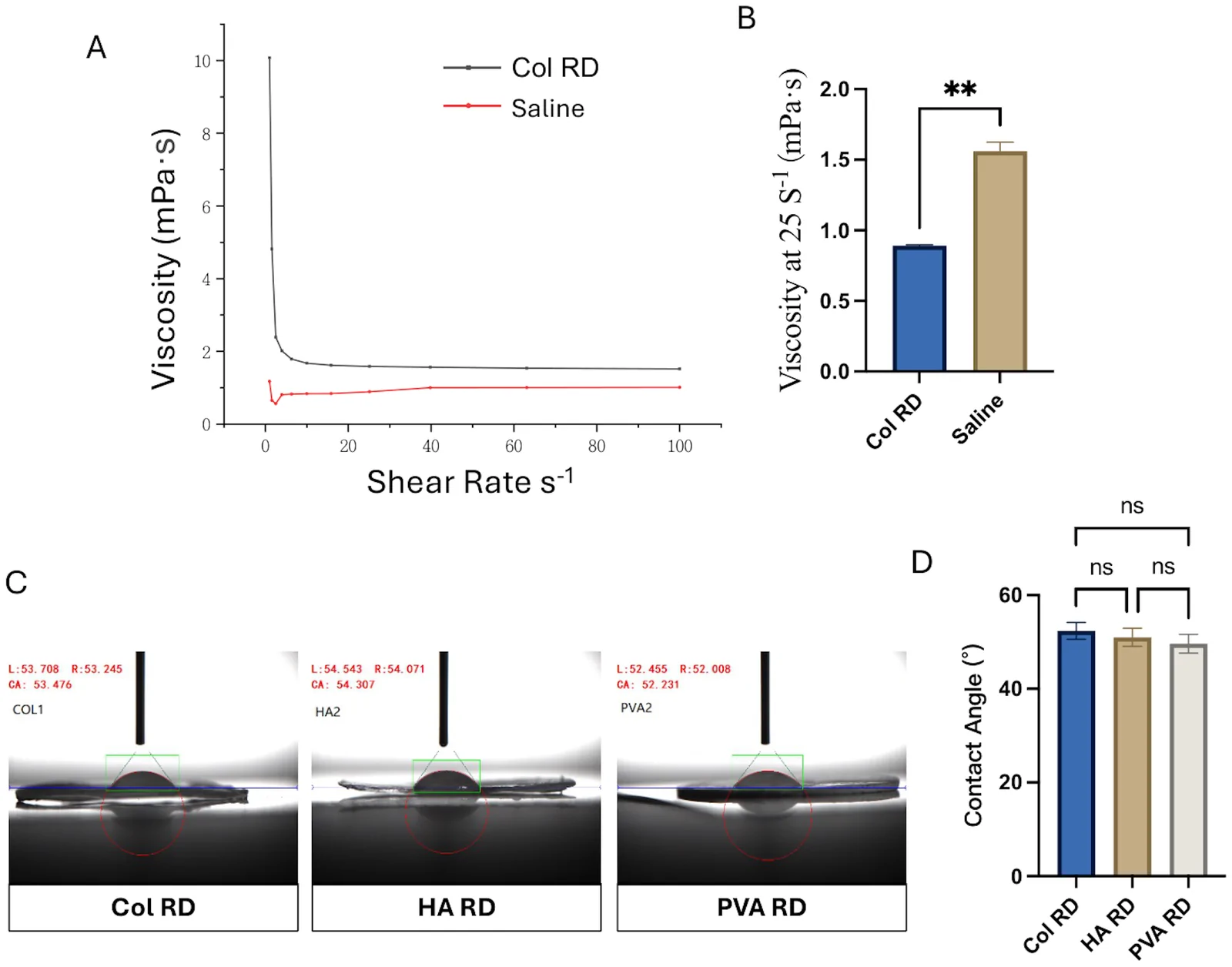
Dynamic viscosity and hydrophilicity of collagen rewetting drops (Col RD). The dynamic viscosity of Col RD exhibited shear-thinning behavior **(A)**, and at a shear rate of 25 s^-^¹, its viscosity was significantly lower than that of physiological saline [**(B)**, n=3, p < 0.01]. **(C)** Contact angle measurement of Col RD, hyaluronic acid rewetting drops (HA RD), and polyvinyl alcohol rewetting drops (PVA RD). **(D)** There was no significant difference in the contact angles measured for Col RD, HA RD, and PVA RD (n=3, p>0.05). ** p < 0.01.

First, the dynamic viscosity was evaluated. The results showed that the recombinant collagen contact lens rewetting solution exhibited shear-thinning behavior, allowing viscosity to decrease during blinking to facilitate ocular surface lubrication (21). At 25 °C and a shear rate of 25 s^-^¹, the physiological saline control group had a dynamic viscosity of 0.89 mPa·s, which is relatively low and insufficient for sustained ocular surface hydration (**Figures 2A, B**). The dynamic viscosity of Col RD was 1.56 mPa·s, higher than saline and close to human tear fluid viscosity during blinking (~1 mPa·s), enabling effective lubrication during eyelid movement and potentially preventing corneal epithelial damage caused by abnormally viscous tear films.

Subsequently, Col RD was compared with two commercially available ophthalmic lubricants, respectively based on sodium hyaluronate and polyvinyl alcohol as the main active ingredients. No significant differences were observed in contact angle measurements among the groups (**Figure 2C**; p > 0.05).

As Col RD contains both recombinant collagen and PVA, the present study evaluated the combined formulation, and the individual contribution of each component could not be determined without collagen-only and PVA-only controls.

### 3.3 Residual protein on RGP lenses after simulated tear exposure and cleaning

Different lubricant formulations (e.g., polymers, viscoelastic agents, humectants) may significantly affect protein deposition, redistribution, and cleaning efficiency on lens surfaces. Artificial tear fluid was prepared to simulate human tears, and contact lenses were incubated in this solution. Protein residue on lens surfaces was compared after treatment with sodium hyaluronate rewetting drops and Col RD, both before and after enzymatic cleaning (22, 23).

**Figure 3:**
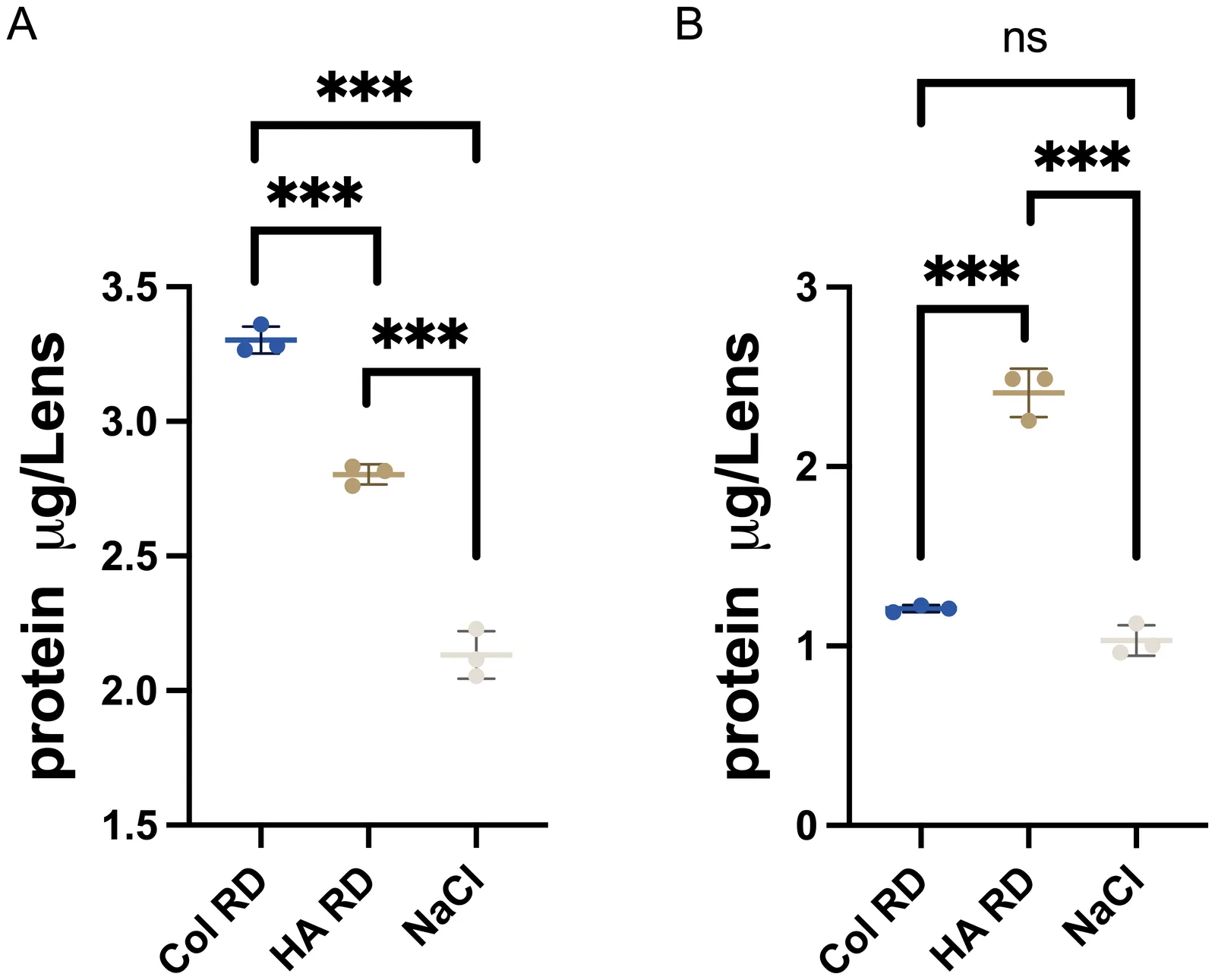
Post-cleaning total residual protein on rigid gas permeable orthokeratology lenses after use of Col RD and HA RD. **(A)** Without enzymatic cleaning, protein residue on lenses after using Col RD was significantly higher than that in the HA RD group and the saline control group. **(B)** With enzymatic cleaning, Col RD group showed lower post-cleaning total residual protein than HA RD group under the present *in vitro* RGP model. (n=3, p < 0.01), and the cleanliness showed no significant difference compared with the blank saline control group (n=3, p > 0.05). *** p < 0.001.

As shown in the **Figure 3A**, after incubation in artificial tear fluid and different rewetting solutions, protein deposition was significantly increased compared with the saline control group. However, after enzymatic cleaning simulating daily lens care procedures, Col RD showed lower residual protein than HA RD after enzymatic cleaning. (**Figure 3B**; p < 0.001), suggesting lower post-cleaning total residual protein under the present *in vitro* RGP lens model. This finding may indicate improved cleanability or reduced post-cleaning residue, but the model does not distinguish initial deposition from cleaning efficiency.

### 3.4 *In vitro* cytocompatibility of Col RD

**Figure 4:**
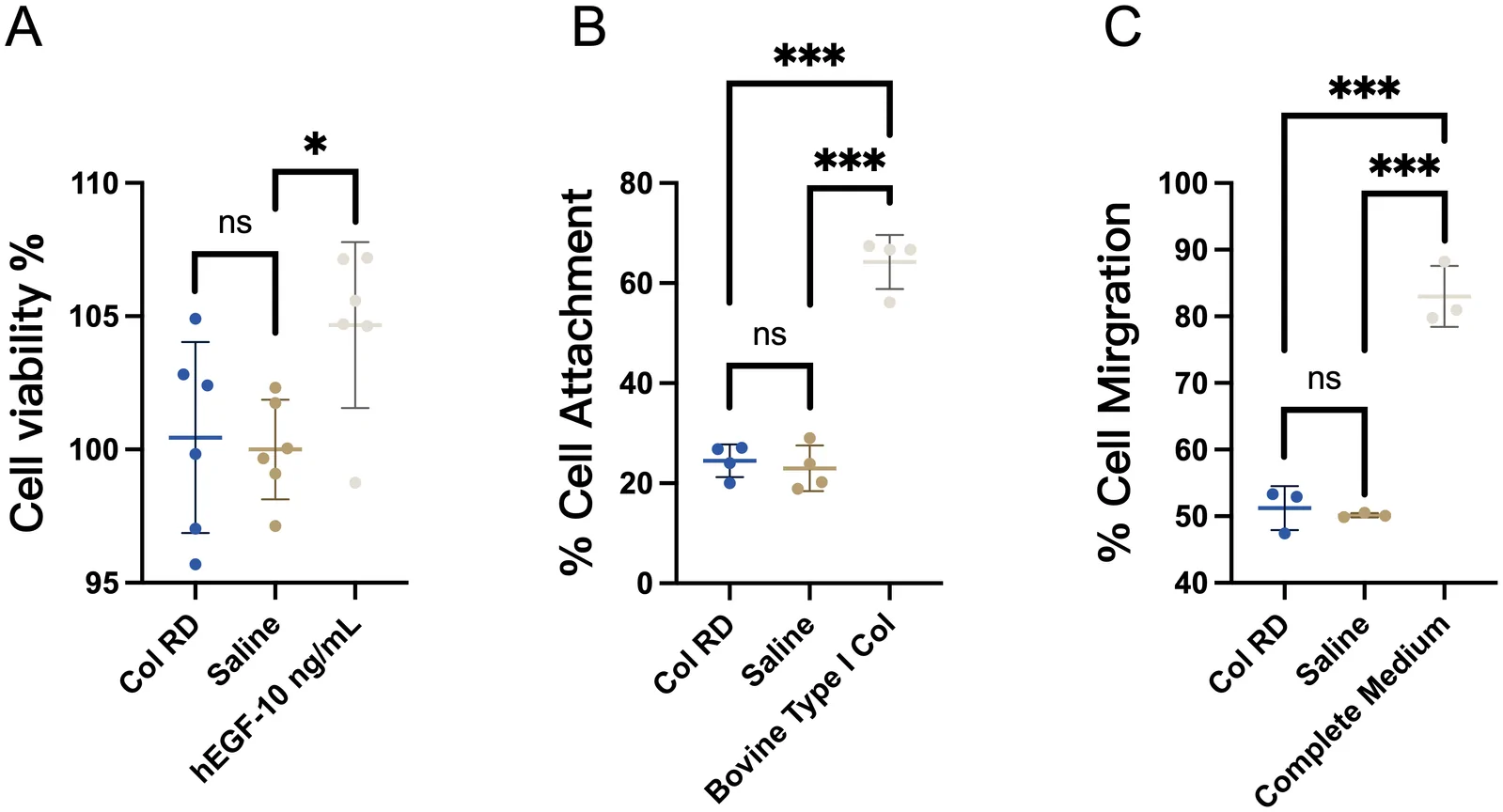
Col RD had no significant effect on the proliferation [**(A)**, n=6], adhesion [**(B)**, n=4], or migration [**(C)**, n=3] of human corneal epithelial cells (HCE-T) (p > 0.05 compared with the saline blank control). *p < 0.05; *** p < 0.001.

As shown in **Figures 4A–C**, Col RD had no significant effects on the viability, adhesion, or migration of human corneal epithelial cells (HCE-T). Compared with the negative control group (saline), no significant difference in cell viability was observed (p > 0.05), while the positive control (hEGF) significantly promoted epithelial cell proliferation (p < 0.05, **Figure 4A**). In the cell adhesion assay, no significant difference was observed between the experimental and control groups (p > 0.05), whereas the positive control (bovine type I collagen) significantly enhanced cell adhesion (p < 0.001) (**Figure 4B**). Similarly, in the cell migration assay, no significant difference was observed between the experimental and negative control groups (p > 0.05), while the complete medium group significantly promoted cell migration (**Figure 4C**; p < 0.001).These results indicate that Col RD did not impair basic corneal epithelial cell behaviors under the present *in vitro* conditions, supporting its cytocompatibility rather than demonstrating a regenerative effect.

### 3.5 Long-term ocular observation

To further evaluate the long-term safety of Col RD, New Zealand rabbits were used as the animal model. Col RD, HA RD, and saline were instilled into both eyes, and ocular irritation, inflammatory responses, and pathological changes were monitored. Histopathology, hematology, and slit-lamp examinations were used to evaluate *in vivo* long-term safety.

**Figure 5:**
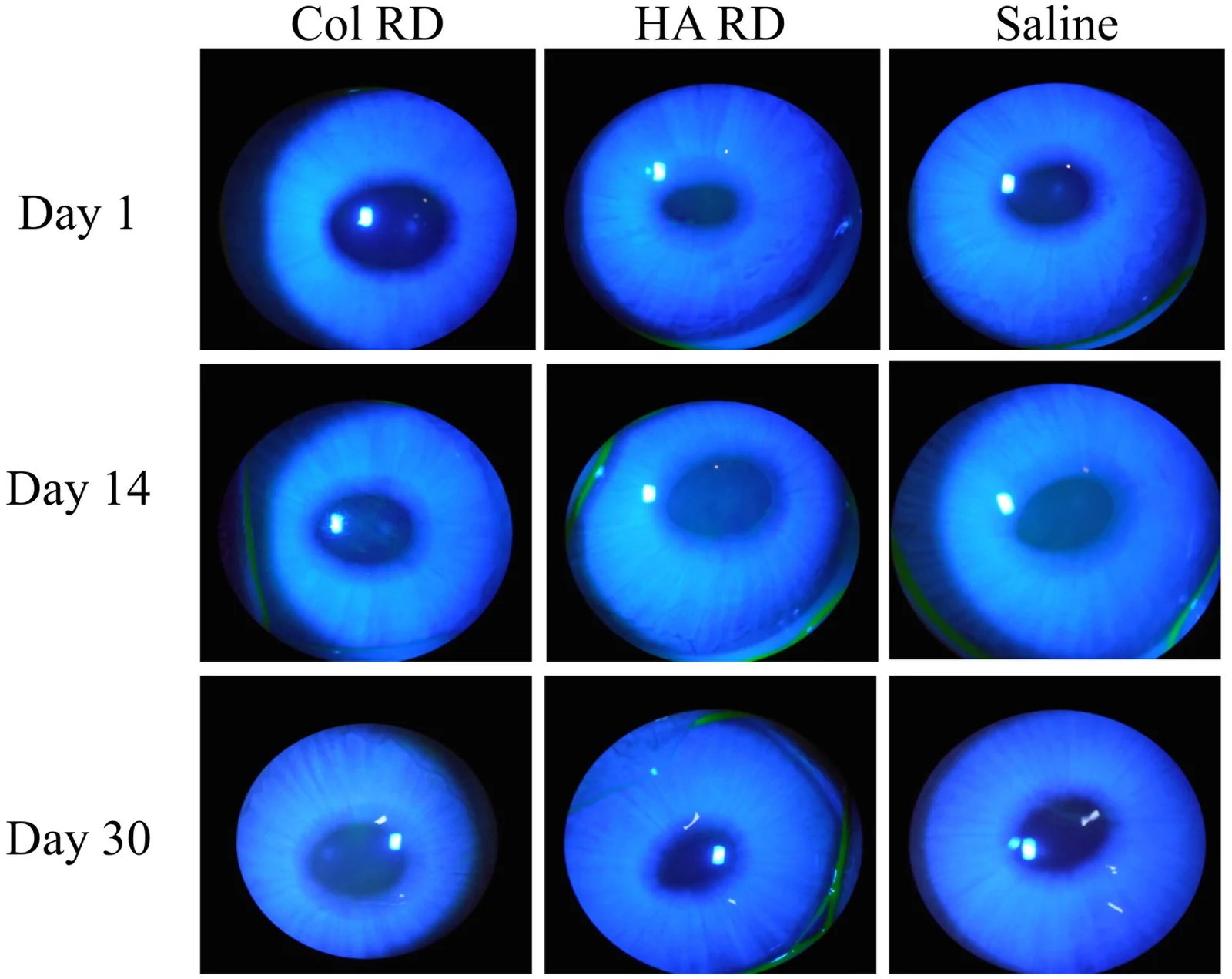
Slit-lamp examination with fluorescein staining after repeated ocular administration. Representative slit-lamp images of rabbit eyes in the Col RD, HA RD, and saline groups on days 1, 14, and 30 after administration. Fluorescein staining was used to evaluate corneal epithelial integrity. No obvious corneal epithelial staining, epithelial defect, corneal opacity, or visible ocular irritation was observed in any group. (n=3 rabbits per group).

Animals were examined and photographed on days 1, 14, and 30 after administration. Fluorescein sodium staining (**Figure 5**), which was used to evaluate corneal epithelial integrity, showed no obvious corneal epithelial staining, epithelial defect, or corneal surface damage throughout the 30-day observation period.

### 3.6 Hematological and serum immunological evaluation after long-term use

After one month of continuous ocular administration, no statistically significant differences were observed in hematological parameters (**Table 3**) among groups (p > 0.05). For serum immunological parameters, most markers showed no statistically significant differences between groups (**Table 4**). IFN-γ was higher in the Col RD group than in the HA RD group (p = 0.0341), whereas no significant difference was observed between Col RD and saline (p = 0.2330). No significant differences were observed for C3a, C5a, IgG, IgM, IL-1β, IL-2, IL-4, IL-5, IL-6, IL-10, IL-12p70, TGF-β, or TNF-α in the planned comparisons involving Col RD (adjusted p > 0.05). These results indicate that the test formulation did not induce measurable changes in routine hematological parameters, and that serum immunological findings were largely comparable among groups under the conditions of this exploratory study.

**Table 3:** Hematological examination results after one month of eye drops administration.

Parameters	Col RD	HA RD	Saline	p Value	p Value
				Col RD vs HA RD	Col RD vs Saline
WBC/(10^9/L)	5.82 ± 1.41	6.12 ± 0.63	6.84 ± 1.55	0.956	0.610
NEU/(10^9/L)	0.94 ± 0.17	1.49 ± 1.09	1.65 ± 0.31	0.586	0.435
LYM/(10^9/L)	4.38 ± 1.20	4.05 ± 0.87	4.55 ± 1.79	0.952	0.987
MON/(10^9/L)	0.42 ± 0.04	0.42 ± 0.06	0.53 ± 0.18	0.879	0.573
EOS/(10^9/L)	0.09 ± 0.02	0.15 ± 0.04	0.12 ± 0.05	0.148	0.607
BAS/(10^9/L)	0.00 ± 0.00	0.00 ± 0.01	0.00 ± 0.00	0.441	1.000
RBC/(10^12/L)	5.30 ± 0.36	5.03 ± 0.14	4.27 ± 0.64	0.738	0.060
HGB/(g/L)	114.33 ± 8.39	108.33 ± 4.51	96.00 ± 11.14	0.678	0.084
HCT/(%)	28.73 ± 3.35	27.07 ± 1.77	23.87 ± 2.81	0.746	0.152
MCV/(fL)	54.10 ± 2.61	53.77 ± 2.37	56.03 ± 1.93	0.822	0.356
MCH/(pg)	21.63 ± 0.21	21.53 ± 0.32	22.60 ± 0.95	0.977	0.194
MCHC/(g/L)	400.33 ± 16.26	401.00 ± 11.14	403.33 ± 3.51	0.997	0.946
PLT/(10^12/L)	254.00 ± 57.09	253.67 ± 14.36	283.67 ± 41.02	0.998	0.679

WBC, white blood cell count; NEU, neutrophil count; LYM, lymphocyte count; MON, monocyte count; EOS, eosinophil count; BAS, basophil count; RBC, red blood cell count; HGB, hemoglobin; HCT, hematocrit; MCV, mean corpuscular volume; MCH, mean corpuscular hemoglobin; MCHC, mean corpuscular hemoglobin concentration; PLT, platelet count; Col RD, recombinant collagen-based rewetting drops; HA RD, hyaluronic acid-based rewetting drops.

**Table 4:** Serum immunological parameter results after one month of eye drop administration.

Parameters	Col RD	HA RD	Saline	p Value	p Value
				Col RD vs HA RD	Col RD vs Saline
C3a (μg/mL)	1.79 ± 0.88	1.93 ± 0.77	1.28 ± 0.70	0.979	0.717
C5a (ng/mL)	48.91 ± 23.91	30.02 ± 11.14	63.04 ± 24.10	0.537	0.695
IgG (mg/mL)	17.81 ± 4.56	9.95 ± 0.74	17.7 ± 9.24	0.311	1.000
IgM (μg/mL)	150.52 ± 65.5	264.29 ± 99.79	169.74 ± 63.58	0.253	0.952
IL-1β (pg/mL)	10.49 ± 4.40	12.04 ± 1.36	12.31 ± 3.34	0.624	0.935
IL-2 (pg/mL)	8.53 ± 3.87	11.49 ± 4.86	6.24 ± 2.61	0.642	0.760
IL-4 (pg/mL)	14.82 ± 10.46	12.09 ± 1.95	15.95 ± 5.61	1.000	1.000
IL-5 (pg/mL)	26.01 ± 15.70	5.97 ± 3.18	9.37 ± 1.38	0.105	0.456
IL-6 (pg/mL)	19.22 ± 10.19	20.2 ± 5.89	23.95 ± 2.95	0.979	0.691
IL-10 (pg/mL)	171.45 ± 115.44	135.02 ± 74.71	229.67 ± 109.81	0.429	0.760
IL-12p70 (pg/mL)	16.6 ± 10.25	14.87 ± 6.2	22.05 ± 8.52	0.967	0.725
TGF-β (pg/mL)	7.13 ± 2.55	7.41 ± 2.67	3.88 ± 1.93	0.655	0.147
TNF-α (pg/mL)	11.34 ± 6.57	11.91 ± 4.76	11.49 ± 4.62	0.991	0.999
IFN-γ (pg/mL)	160.5 ± 90.50	30.31 ± 19.2	52.85 ± 7.81	0.034	0.233

C3a, complement component 3a; C5a, complement component 5a; IFN-γ, interferon gamma; IgG, immunoglobulin G; IgM, immunoglobulin M; IL, interleukin; IL-1β, interleukin-1 beta; IL-2, interleukin-2; IL-4, interleukin-4; IL-5, interleukin-5; IL-6, interleukin-6; IL-10, interleukin-10; IL-12p70, interleukin-12 p70; TGF-β, transforming growth factor beta; TNF-α, tumor necrosis factor alpha; Col RD, recombinant collagen-based rewetting drops; HA RD, hyaluronic acid-based rewetting drops.

The corneal epithelial layer forms a tight barrier that restricts the penetration of macromolecules such as recombinant collagen and sodium hyaluronate. In addition, instilled solutions on the ocular surface are rapidly diluted by tears and removed through blinking. However, a fraction of topically instilled material may enter the nasolacrimal drainage pathway; therefore, systemic exposure is expected to be low rather than absent.

These exploratory findings support the preliminary repeated-dose ocular tolerability of Col RD and suggest that no obvious systemic hematological or immune abnormalities were detected under the present experimental conditions. Systemic exposure was not directly quantified in this study, and the relatively small sample size should be considered when interpreting these exploratory safety findings.

### 3.7 Corneal histology: HE and Masson staining

To evaluate the effects of one-month continuous ocular administration on corneal morphology, inflammatory response, and fibrosis, HE and Masson staining were performed.

**Figure 6:**
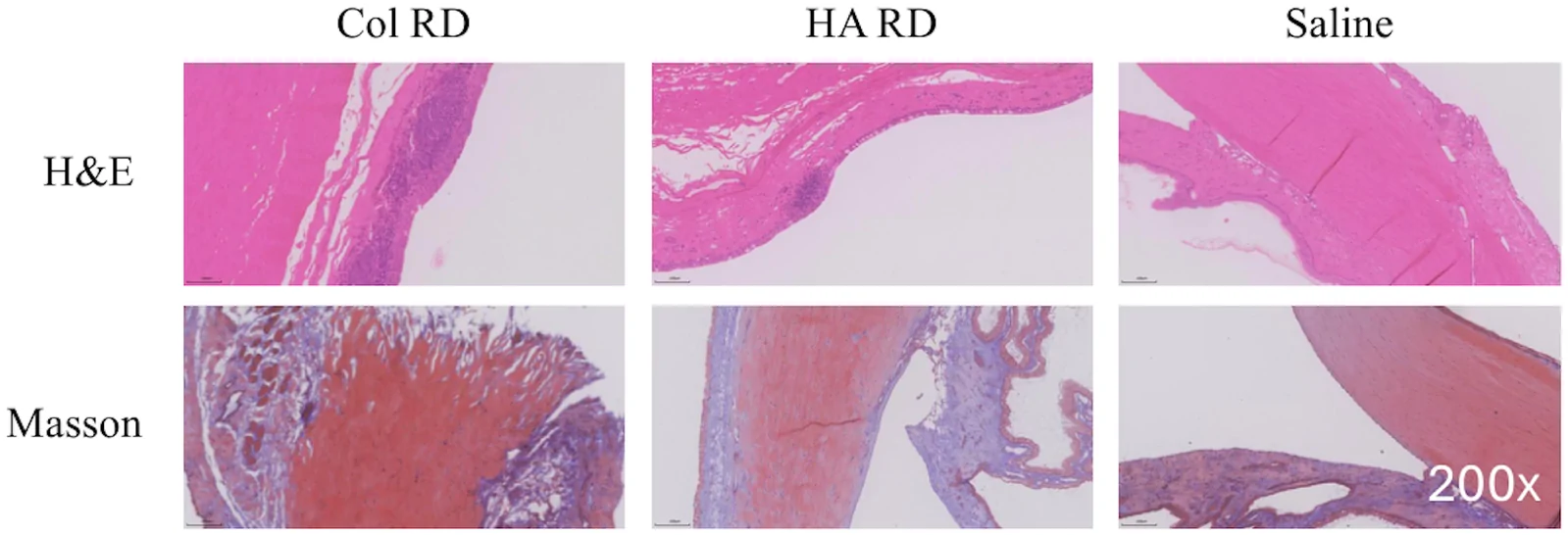
Histological H&E and Masson staining after one month of eye drop administration. In all groups, no tissue necrosis, no evident neovascularization or fibrosis, no macrophage or histiocyte infiltration, no multinucleated giant cell reaction, and no adipose tissue infiltration were observed (n=3 rabbits per group, upper, 200×). Masson staining showed that collagen fibers in the extracellular matrix were densely and uniformly arranged in all groups, with no abnormal substance deposition (n=3 rabbits per group, lower, 200×).

As shown in **Figure 6** (H&E staining, 200x), all groups showed no necrosis, no neovascularization, and no fibrosis in corneal tissues. No infiltration of macrophages or histiocytes, no multinucleated giant cell reaction, and no lipid infiltration were observed. Mild lymphocytic infiltration was observed in the superficial dermis of the eyelid (chronic inflammation localized to eyelid tissue, with no functional impact on the eye). There were no significant differences in histological scoring between the Col RD and HA RD groups, both of which represented mild physiological responses without pathological damage. The blank group showed slightly higher scores than the Col RD and HA RD groups.

These findings indicate that neither Col RD nor HA RD caused ocular irritation. Moreover, both recombinant collagen and sodium hyaluronate may have modulatory effects on ocular surface responses, potentially alleviating discomfort associated with long-term contact lens wear compared with saline.

As shown in **Figure 6**, Masson staining (200x) after one month showed dense and uniformly arranged collagen fibers in all groups, with no abnormal deposition. Therefore, Col RD did not induce abnormal changes in corneal tissue after one month of use.

### 3.8 Sirius red staining of corneal tissue

Given that recombinant collagen is a macromolecular polypeptide with potential biological activity affecting collagen composition and distribution, Sirius Red staining was further performed to assess changes in corneal collagen after long-term use.

**Figure 7:**
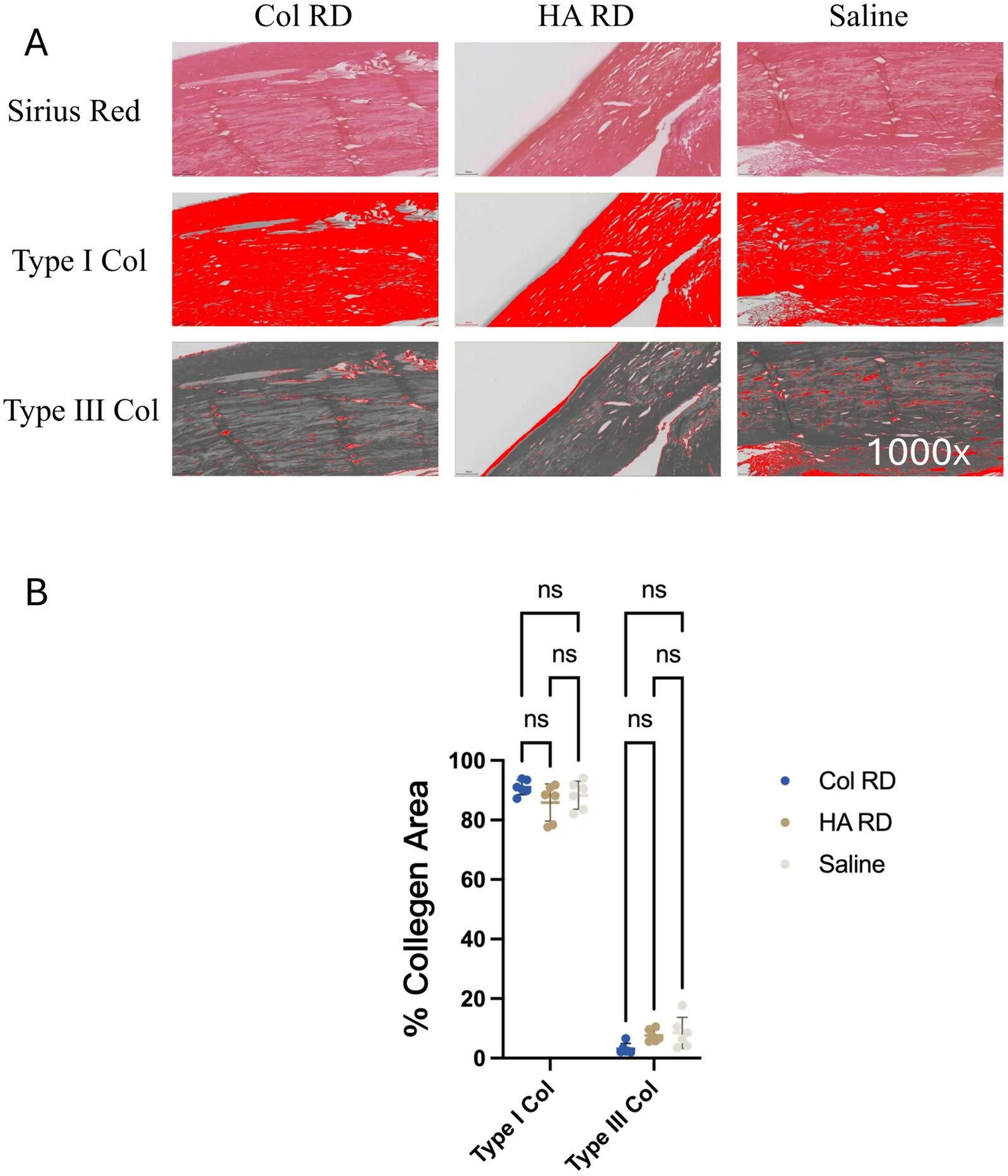
Quantitative analysis of Sirius Red staining after one month of eye drop administration. **(A)** The Col RD group showed no abnormal collagen over-deposition, no disruption in collagen ratio, and no pathological fibrotic changes. The levels of collagen proliferation and maturation were comparable to those in the saline group (1000×). **(B)** There were no significant differences in the areas and proportions of type I and type III collagen among the Col RD, HA RD, and saline groups (n=6 eyes per group of 3 rabbits, p > 0.05).

The results showed no significant differences in the area or proportion of type I and type III collagen between the Col RD, HA RD, and saline groups (**Figure 7**). Collagen proliferation and maturation levels in the Col RD group were comparable to those in the saline group.

No abnormal collagen over-deposition, disruption of collagen ratio, or pathological fibrotic changes were observed. These findings further demonstrate that the recombinant type III collagen used in this study functions primarily as a physical lubricating and moisturizing agent without additional biological activity.

## 4 Discussion

Collagen has a long history of application in humans. However, due to limitations in extraction purity, batch-to-batch variability, and allergenic risk, it has been mainly used in severe wound conditions such as burns and tissue defects (24, 25). Concerns regarding its long-term safety have persisted.

Recombinant collagen, through sequence design combined with precise, high-efficiency fermentation and purification processes, enables controllable amino acid sequences, significantly improved purity, and elimination of batch-to-batch variability (26). However, because recombinant collagen sequences still differ to some extent from native sequences, its safety must be evaluated separately according to specific sequences and application scenarios (24, 27).In the present study, the recombinant type III collagen sequence was evaluated as a human-sequence-derived protein component for an aqueous contact lens rewetting formulation, rather than as a general substitute for native collagen materials.

The collagen used in this study was designed with particular emphasis on maintaining a hydrophilic sequence profile and avoiding exogenous affinity tags or non-human functional domains. Although sequence selection may reduce certain theoretical concerns related to non-human components, immunogenic epitope removal and hydrolytic stability were not directly mapped in this study. Therefore, these design-related statements should be interpreted as formulation rationale, while the safety conclusion is based on the *in vitro* cytocompatibility and repeated-dose ocular safety data presented here.

Our study demonstrates that the formulated rewetting drops provide good hydrophilicity and exhibit shear-thinning rheological behavior, which is consistent with the intended use of Col RD as an aqueous lubricating formulation for contact lens rewetting. However, because the formulation contains both recombinant collagen and 0.5% PVA, and PVA itself is a lubricant and viscosity modifier, the present study cannot determine the individual contribution of each component to the rheological profile or lubricating performance. Accordingly, the present study should be interpreted as demonstrating the feasibility of incorporating recombinant type III collagen into a lubricating ophthalmic formulation, rather than proving the superiority of recombinant collagen itself over existing lubricant components. Further studies using collagen-only, PVA-only, and other comparator formulations are needed to determine the independent contribution of recombinant collagen to lubrication, lens compatibility, and ocular tolerability.

Furthermore, a one-month long-term ocular administration study showed no evident local irritation, structural damage, or abnormal hematological or serum immunological changes under the conditions tested. These findings support preliminary ocular tolerability of Col RD after repeated topical administration, but they should not be interpreted as proof of absent systemic exposure or complete systemic safety, because a fraction of topically instilled material may enter the nasolacrimal drainage pathway. In addition to ocular tolerability, compatibility between contact lens lubricants and lenses themselves is also an important consideration, as protein residues on lenses may affect optical clarity, comfort, microbial adhesion, and ocular surface responses.

Given that recombinant collagen is the primary component of our formulation, its residual behavior on contact lenses warranted careful investigation. We prepared a simulated tear fluid containing inorganic salts, proteins, and lipids and an RGP lens cleaning model to evaluate total residual protein on lens surfaces before and after cleaning. Without enzymatic cleaning, Col RD increased protein residue on lenses. After enzymatic cleaning, which is routinely used for RGP lenses, the Col RD group exhibited significantly lower residual protein levels than the HA RD group.

This suggests that under the present *in vitro* RGP lens model, Col RD may be associated with lower post-cleaning protein residue than the HA-based comparator. However, this result should be interpreted cautiously. NanoOrange quantification measured total residual protein and could not distinguish residual recombinant collagen from tear proteins such as albumin or lysozyme. The post-cleaning result may also reflect easier removal rather than reduced initial deposition. Therefore, the finding is better described as a difference in residual protein or cleanability, not direct evidence that Col RD prevents protein deposition.

The mechanisms underlying the different post-cleaning residues remain unclear. Previous studies have shown that contact lens surface properties, tear proteins, lens materials, and cleaning methods can all influence protein deposition and removal (28–31).Although polysaccharide-tear protein interactions may contribute to residue behavior, this study did not directly evaluate hyaluronic acid-lysozyme complex formation, lysozyme activity, or tear antimicrobial function. These possibilities should be regarded only as hypotheses for future investigation.

The *in vitro* cell assays showed that Col RD did not significantly affect the viability, adhesion, or migration of HCE-T cells compared with the corresponding controls. These results indicate that the recombinant collagen lubricant does not significantly promote or inhibit proliferation, adhesion, or migration of corneal epithelial cells. In the revised interpretation, these assays are framed primarily as cytocompatibility and safety assessments, rather than as evidence of epithelial repair or regenerative activity. The MTT assay evaluated cell growth on a pre-coated surface rather than continuous direct exposure, which should be considered when interpreting the results. The adhesion assay was also a simplified *in vitro* model and may not fully reproduce epithelial attachment behavior on the ocular surface.

The rabbit study further supports the ocular tolerability of Col RD after repeated topical administration. No abnormal ocular morphology, corneal epithelial staining, histological damage, fibrosis, or abnormal collagen distribution was observed during the 30-day observation period. Because only 3 rabbits were included in each group, the statistical analyses of the animal study should be considered exploratory. The absence of statistically significant differences among groups should not be interpreted as definitive evidence of biological equivalence, but rather as preliminary evidence that no obvious adverse ocular or systemic findings were detected under the present experimental conditions. Nevertheless, this animal study evaluated repeated-dose ocular safety, not contact lens rewetting efficacy, because rabbits did not wear contact lenses. Thus, the *in vivo* findings cannot directly demonstrate improved comfort, lubrication, or rewetting performance during actual lens wear. The *in vitro* lens deposition model also had a translational limitation because it used 2 mL of artificial tear fluid and 20 µL of rewetting drops, which does not reflect the much smaller and dynamically changing tear volume on the ocular surface after instillation. Thus, the findings should be interpreted as a simplified *in vitro* assessment of post-cleaning protein residue rather than a direct simulation of the *in vivo* tear/drop volume relationship.

From a translational perspective, the present findings should be regarded as part of a preliminary biological safety evaluation. Further product development should follow a risk-based ISO 10993 biological evaluation framework, with additional tests selected according to the final product classification, contact type, exposure duration, and intended clinical use.

In addition to biological safety, the scalability and manufacturing feasibility of the formulation are important considerations for its future clinical translation. Compared with chemically synthesized collagen-mimetic materials, recombinant collagen production through engineered biological systems provides advantages in sequence programmability, manufacturing consistency, and scalability (32). Although current production costs remain influenced by fermentation yield and downstream purification processes, continued optimization of recombinant expression systems and manufacturing platforms may contribute to future cost reduction and broader biomedical applications (33).

Several limitations should be acknowledged. First, only rigid gas-permeable contact lenses were evaluated, so the results should not be extrapolated to soft hydrogel or silicone hydrogel lenses. Second, the artificial tear model used 2 mL of tear solution and 20 µL of rewetting drops, which does not reproduce the *in vivo* tear/drop volume relationship. Third, rheological measurements were performed at 25 °C rather than at ocular surface or physiological temperature, and temperature-dependent changes in viscosity were not evaluated. Fourth, collagen-only and PVA-only controls were not included. Fifth, the animal study used a small sample size, and no formal sample size calculation was performed, the statistical analyses of the rabbit study should be considered exploratory and should not be used to infer definitive biological equivalence among groups. Finally, clinical wearing comfort, long-term formulation stability, preservative efficacy, endotoxin level, batch-to-batch reproducibility, and ocular microbiome effects were not directly assessed. These issues should be addressed in future studies.

In summary, this study provides preliminary evidence supporting the physicochemical feasibility, *in vitro* cytocompatibility, RGP lens post-cleaning residue profile, and repeated-dose ocular tolerability of recombinant type III collagen-based contact lens rewetting drops. The findings support the feasibility of incorporating recombinant type III collagen into a PVA-containing lubricating ophthalmic formulation for contact lens rewetting. However, the present study does not establish the independent superiority of recombinant collagen over existing lubricant components, and further studies are required to determine its specific contribution within the combined formulation. Clinical efficacy and broader lens compatibility remain to be established.

## Data Availability

The raw data supporting the conclusions of this article will be made available by the authors, without undue reservation.
